# Somatic Mutation Detection in Tumor Tissue and Matched Cell-Free DNA Using PCR-Based Methods in Pancreatic Cancer Patients Undergoing Upfront Resection

**DOI:** 10.3390/ijms26178518

**Published:** 2025-09-02

**Authors:** Hana Zavrtanik Čarni, David Badovinac, Tanja Blagus, Katja Goričar, Branislava Ranković, Alenka Matjašič, Andrej Zupan, Aleš Tomažič, Vita Dolžan

**Affiliations:** 1 Department of Abdominal Surgery, University Medical Centre Ljubljana, 1000 Ljubljana, Slovenia; 2 Faculty of Medicine, University of Ljubljana, 1000 Ljubljana, Slovenia; 3 Institute of Biochemistry and Molecular Genetics, Faculty of Medicine, University of Ljubljana, 1000 Ljubljana, Slovenia; 4 Institute of Pathology, Faculty of Medicine, University of Ljubljana, 1000 Ljubljana, Slovenia; branislava.rankovic@mf.uni-lj.si (B.R.); alenka.matjasic@mf.uni-lj.si (A.M.); andrej.zupan@mf.uni-lj.si (A.Z.)

**Keywords:** somatic mutation detection, cell-free DNA, liquid biopsy, digital PCR, pancreatic cancer

## Abstract

Somatic mutations in *KRAS* and *TP53* are among the most common genetic alterations in pancreatic ductal adenocarcinoma (PDAC). Advances in PCR-based technologies now enable the detection of these mutations in tumor tissue and cell-free DNA (cfDNA), providing a minimally invasive approach to assess tumor burden. However, in resectable PDAC, circulating tumor DNA (ctDNA) may represent less than 0.1% of total cfDNA, requiring highly sensitive detection methods. The aim of our study was to assess two PCR-based assays—competitive allele-specific PCR (castPCR) and digital PCR (dPCR)—for detecting selected somatic mutations in tumor tissue, cfDNA, and extracellular vesicle-associated DNA (EV-DNA) from plasma. Matched primary tumor and preoperative plasma samples were collected from 50 patients undergoing upfront resection for PDAC. CastPCR was used for detecting selected *KRAS*, *TP53*, *SMAD4,* and *CDKN2A* mutations in tumor DNA. Additionally, dPCR was used to analyze *KRAS* and *TP53* mutations in tumor DNA as well as cfDNA and EV-DNA. The concordance between both platforms was 71.4% for *KRAS* p.G12D and 58.3% for the analysis of *TP53* p.R273H mutations in tumor tissue. However, dPCR detected these mutations in an additional 28.6% and 39.6% of samples, respectively. In cfDNA, dPCR identified *KRAS* p.G12D in 10.2% and *TP53* p.R273H in 2.0% of samples. Mutation detection in EV-DNA was limited by low DNA yield. Both platforms proved effective for tumor DNA analysis, with dPCR offering greater sensitivity. Somatic mutation detection from liquid biopsy using dPCR further supports its potential utility in the preoperative setting.

## 1. Introduction

Pancreatic ductal adenocarcinoma (PDAC) is an aggressive malignancy with a dismal prognosis, ranking among the leading causes of cancer-related mortality worldwide [1]. Despite surgical resection offering the only potential for long-term survival, most patients experience recurrence within two years, even when no clinical or radiological signs of metastasis are evident at the time of surgery [2,3,4]. This highlights the need for more sensitive biomarkers to improve patient selection and enable personalized treatment strategies.

Somatic mutations in *KRAS* and *TP53* are among the most common genetic alterations in PDAC, present in over 90% and 70% of cases, respectively [5,6]. These mutations represent key drivers of tumorigenesis but can also serve as valuable molecular markers for diagnosis, prognosis, and potential therapeutic stratification [5,6,7,8]. Traditionally, such mutations are identified in tumor tissue; however, spatial heterogeneity and sampling limitations can compromise the accuracy of tissue-based molecular profiling [8,9].

The emergence of liquid biopsy with an assessment of circulating nucleic acids, particularly circulating tumor DNA (ctDNA) within cell-free DNA (cfDNA) or extracellular vesicle-associated DNA (EV-DNA), offers a promising, minimally invasive approach to overcome these limitations. Nevertheless, ctDNA can be extremely scarce in early-stage, resectable PDAC—often comprising less than 0.1% of total cfDNA—demanding highly sensitive detection technologies [10,11,12].

In this study, we assessed the performance of competitive allele-specific TaqMan PCR (castPCR) and digital PCR (dPCR) for the detection of selected *KRAS*, *TP53*, *SMAD4*, and *CDKN2A* mutations in matched primary tumor tissue and preoperative plasma samples from patients undergoing upfront surgical resection for PDAC. Furthermore, we explored the utility of dPCR for detecting *KRAS* and *TP53* mutations in cfDNA and EV-DNA, evaluating the feasibility of liquid biopsy as a complementary tool in the preoperative management of PDAC.

## 2. Results

### 2.1. Patients’ Characteristics

We analyzed tissue and preoperative plasma samples from 50 patients with PDAC who were treated with surgical resection. Median patient age was 70 years, and 54% of patients were male. The majority of patients had stage 2 or stage 3 disease (52.0% and 28.0%, respectively). In three patients, metastatic disease was identified during the final histologic examination of resected specimens, which had not been detected intraoperatively or during frozen section analysis. This included two patients with peritoneal deposits and one patient with carcinomatous deposits in the spleen. The patients’ clinical characteristics are summarized in Table 1.

### 2.2. Frequency of Genetic Mutations in Primary Tumor and Plasma Samples

#### 2.2.1. Results of castPCR Analyses

**Figure 1:**
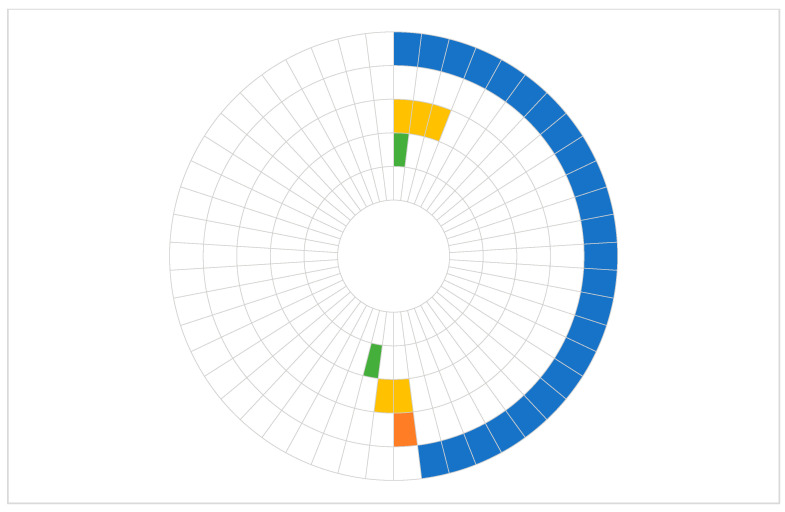
Somatic mutations detected by castPCR in tumor DNA samples from 50 patients (from outside inwards: *KRAS* p.G12D (blue), *KRAS* p.G12V (orange), *TP53* p.R273H (yellow), *CDKN2A* p.H83Y (green), *SMAD4* p.R445* (not detected)).

Using castPCR analysis of tumor DNA samples, the *KRAS* p.G12D mutation was most frequently identified (48.0% of patients) with a median %mutation of 7.0% (IQR 5.3–13.7%). Other selected mutations were detected less frequently, as shown in Table 2. In the majority of patients, only one mutation was detected, while four patients demonstrated the presence of multiple mutations. Two patients harbored both *KRAS* p.G12D and *TP53* p.R273H: one had *KRAS* p.G12V and *TP53* p.R273H, and the other patient had *KRAS* p.G12D, *TP53* p.R273H, and *CDKN2A* p.H83Y (Figure 1).

#### 2.2.2. Results of dPCR Analyses

*KRAS* p.G12D and *TP53* p.R273H mutations were selected for further analyses with dPCR, as they were the ones most frequently detected by castPCR. Mutations found in primary tumor samples were then investigated in matched plasma samples.

dPCR analysis showed that a total of 95.9% and 75.5% of primary tumor samples harbored the *KRAS* p.G12D mutation, with a median %mutation of 15.2% (IQR 0.2–26.2%) and 16.7% (IQR 10.9–34.5%), when the %mutation cutoff for a positive reaction was set at >0% and >0.1%, respectively. In matched preoperative plasma samples, *KRAS* p.G12D mutations were identified in 32.7% and 10.2%, with a median %mutation of 0.1% (IQR 0.0–0.2) and 0.2% (IQR 0.2–0.2) for the same cutoff thresholds. In contrast, *TP53* p.R273H mutations were identified in 93.8% and 47.9% of primary tumor samples with a markedly lower median %mutation of 0.1% (IQR 0.1–0.1) and 0.1% (IQR 0.1–0.2), at >0% and >0.1% cutoffs, respectively. The mutation was present in 8.2% and 2.0% of matched plasma samples at the respective cutoffs (Table 3).

**Figure 2:**
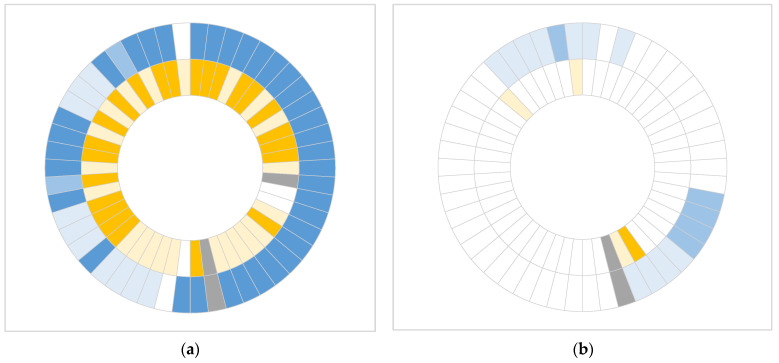
Somatic mutations detected by dPCR in primary tumor (**a**) and preoperative cfDNA (**b**) samples from 50 patients (from outward inwards: *KRAS* p.G12D (blue), *TP53* p.R273H (yellow)). Dark blue >1%, medium blue 0.1–1%, light blue <0.1%; dark yellow >0.1%, light yellow <0.1%; gray not determined.

In all but five patients, both mutations (*KRAS* p.G12D and *TP53* p.R273H) were detected in the primary tumor samples. Among these five patients, three harbored only the *KRAS* p.G12D mutation, while two had only the *TP53* p.R273H mutation. In preoperative cfDNA samples, both mutations were detected in only three patients. *KRAS* p.G12D alone was identified in 13 patients, and *TP53* p.R273H alone in one patient. No mutant cfDNA was detected in the remaining 32 patients (Figure 2). In three primary tumor samples, mutation detection was unsuccessful due to higher %mutation found in matched normal tissue DNA compared to the corresponding primary tumor DNA, rendering the results unreliable. Two reactions in cfDNA samples were considered invalid due to an insufficient number of wild-type reference signals (138 and 10 copies) below the required threshold of 200 copies.

*KRAS* p.G12D mutation was not detected in any EV-DNA samples; however, all samples contained fewer than 200 wild-type allele copies, indicating insufficient DNA input to consider the reactions analytically valid. Therefore, further analysis of additional samples was not pursued.

### 2.3. Comparative Analysis of Mutation Detection with castPCR and dPCR

The detection of *KRAS* p.G12D and *TP53* p.R273H in tissue samples was compared between castPCR and dPCR analyses. For the *KRAS* p.G12D mutation, the results of castPCR and dPCR analysis were concordant in 25 of 49 patients (51.0%) and 35 of 49 patients (71.4%), depending on the %mutation cutoff for a positive reaction in dPCR (>0% and >0.1%, respectively). However, dPCR displayed somewhat better sensitivity; the mutation was discovered upon dPCR but not castPCR analysis in 24 of 49 patients (49.0%) and 14 of 49 patients (28.6%) for the same cutoff thresholds (Table 4).

**Figure 3:**
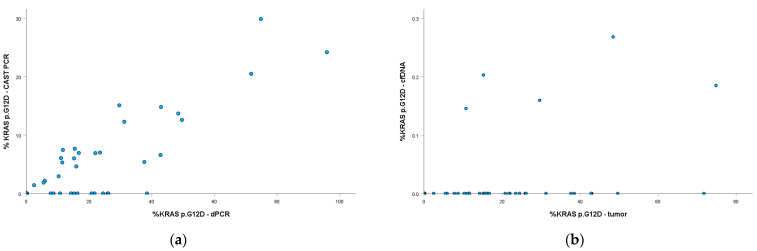
*KRAS* p.G12D mutation detection: (**a**) Correlation between the %mutation detected by castPCR and dPCR in tumor DNA samples (Spearman’s rho = 0.670, *p* < 0.001); (**b**) Correlation between the %mutation detected by dPCR in tumor DNA and cfDNA samples (Spearman’s rho = 0.292, *p* = 0.044). Only values >0.1% were considered positive.

There was a strong correlation between the percentage of *KRAS* p.G12D mutations determined in tumor DNA samples by the two methods, with a Spearman’s rho of 0.666 (*p* < 0.001) and 0.670 (*p* < 0.001) when %mutation of >0% and >0.1% were considered for a positive reaction in dPCR, respectively (Figure 3).

For the *TP53* p.R273H mutation, the two methods demonstrated concordance in 6 of 48 patients (12.5%) and 28 of 48 (58.3%) patients, depending on the %mutation cutoff for a positive reaction in dPCR (>0% and >0.1%, respectively). Similarly, dPCR exhibited greater sensitivity, detecting the mutation in 41 of 48 patients (85.4%) and 19 of 48 patients (39.6%) for the same cutoff thresholds, whereas castPCR failed to detect it. On the other hand, in one patient, the mutation was identified exclusively by castPCR (Table 5).

### 2.4. Comparative Analysis of Mutation Detection in Tissue and Plasma Samples with dPCR

**Figure 4:**
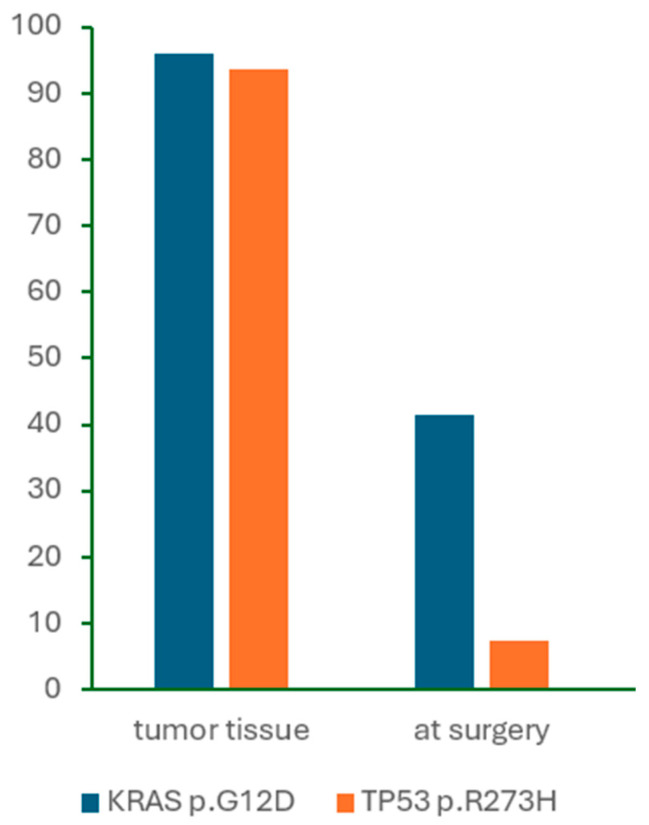
The prevalence of *KRAS* p.G12D and *TP53* p.R273H mutations detected by dPCR in tumor DNA and cfDNA samples.

We also compared *KRAS* p.G12D and *TP53* p.R273H mutation detection by dPCR in tumor DNA and cfDNA samples. The prevalence of detected mutations in the two samples is illustrated in Figure 4.

We observed some correlation only between the *KRAS* p.G12D %mutation detected by dPCR in tumor DNA and cfDNA samples (Spearman’s rho = 0.292, *p* = 0.044) when a >0.1% cutoff was used for a positive reaction (Figure 3b), but not with the >0% cutoff (Spearman’s rho = 0.230, *p* = 0.115). Due to the low number of positive reactions in cfDNA samples, no relevant correlation was observed between the %mutation of *TP53* p.R273H identified in tumor DNA and cfDNA samples (Spearman’s rho = −0.230, *p* = 0.121 and Spearman’s rho = −0.134, *p* = 0.368 when >0% and >0.1% cutoffs were used for a positive reaction).

## 3. Discussion

In this study, we demonstrated that both castPCR and dPCR approaches are effective in detecting common somatic mutations in primary tumor tissue of patients with resectable PDAC. However, dPCR showed superior sensitivity, identifying mutations in a substantial proportion of samples that were negative by castPCR. Furthermore, dPCR was also effective in detecting mutations in cfDNA despite low ctDNA levels in early-stage PDAC.

Our results are concordant with the well-established prevalence of *KRAS* alterations in PDAC. Large-scale studies consistently report *KRAS* mutations in approximately 90–95% of PDAC tumors; however, multiplex dPCR or sequencing methods that report the combined prevalence of various hotspot mutations within the *KRAS* gene are usually used [5,6,13,14,15]. We detected a single *KRAS* mutation (*KRAS* p.G12D) in 48% and 75.5% of primary tumor samples with castPCR and dPCR, respectively. An even higher percentage of positive samples was detected with dPCR when any mutation percentage above 0%, rather than a threshold of >0.1%, was considered indicative of a positive result. Similarly, *TP53* mutations are key drivers in PDAC pathogenesis and are frequently observed alongside *KRAS* alterations, with prevalence estimates around 50–75% [5,6,13]. In our study, *TP53* mutations were detected in only 10% of tumor samples using castPCR, whereas dPCR identified mutations in 47.9% of tumor samples. This discrepancy likely reflects the methodological differences in prior studies, as many of these employed next-generation sequencing or multiplexed assays capable of detecting a broader spectrum of *TP53* variants. In contrast, *CDKN2A* and *SMAD4* mutations are less common and more heterogeneous, typically lacking hotspot sites and including diverse mechanisms of inactivation [13]. Therefore, in our cohort, selected CDKN2A and *SMAD4* mutations were discovered in two and none of the patients, respectively.

The concordance of castPCR and dPCR approaches in mutation detection in primary tissue samples was 71.4% for *KRAS* and 58.3% for *TP53*, although dPCR was able to detect mutations missed by castPCR. The concordance depended greatly on the choice of the cutoff level. We applied two different thresholds to define a positive dPCR result to distinguish between the more sensitive detection threshold (>0%) and a more robust quantification (>0.1%). This approach is in line with the Minimum Information for Publication of Digital PCR Experiments (dMIQE) 2020 guidelines, which emphasize the importance of separating qualitative detection from quantitative reporting [16]. While both approaches enable detection of mutations at frequencies as low as 0.1%, the concordance between them was much lower (51.0% for KRAS and 12.5% for *TP53*), when any %mutation above 0% was considered positive in dPCR. This further highlights the ability of dPCR to identify very low-level mutations beyond the detection limit of castPCR. However, in one tissue sample, a *TP53* p.R275H mutation was detected by castPCR but not by dPCR. Although dPCR generally provides greater analytical sensitivity than castPCR, it has been reported that some castPCR assays, including *TP53* p.R275H, may exhibit higher background at higher DNA inputs compared to dPCR [17]. Another possible reason may be that the primary tumor DNA was extracted from formalin-fixed paraffin-embedded (FFPE) samples. Formalin fixation may lead to DNA damage, and while the blocker-based enriched amplification may result in amplification above the threshold in castPCR, more stringent dPCR will not amplify such preanalytical artifacts [18].

In contrast to primary tumor analysis, the detection of *KRAS* mutations in cfDNA was markedly lower in our study. Numerous studies have demonstrated that *KRAS* mutations are detectable in only a subset of cfDNA samples, with reported detection rates ranging from 10% to 50%, depending on disease stage and analytical sensitivity [14,19,20]. None of the patients in our study exhibited clinical or radiographic evidence of distant metastases at the time of sample collection. Although our cohort included three patients who were ultimately found to have metastatic disease, this was not discovered before the final histopathologic examination. Among these three patients, the *KRAS* p.G12D mutation was detected in the preoperative plasma of one patient only, with a %mutation of 0.20%. Furthermore, no clear correlation was observed between mutation burden in the primary tumor and corresponding perioperative cfDNA samples, suggesting that a higher mutation frequency in tumor tissue does not necessarily increase the likelihood of mutation detection in plasma.

We also attempted to assess mutation detection in EV-DNA, but the DNA yield was insufficient for meaningful analysis. This is consistent with previous findings that although EV-DNA can be a promising source of tumor-derived nucleic acids, its utility remains constrained by low concentrations in plasma and more complex isolation and characterization procedures [21,22]. Interestingly, some studies have reported comparable sensitivity in mutation detection between EV-DNA and cfDNA, suggesting that standard cfDNA extraction methods may already capture a portion of EV-DNA. Consequently, cfDNA analysis may remain a more efficient and cost-effective approach for plasma mutation detection [23,24,25].

This study has several limitations that should be acknowledged. First, the relatively small sample size of 50 patients may limit the statistical power and generalizability of the findings, and validation in larger, independent cohorts is warranted. Second, the extremely low abundance of ctDNA in early-stage, resectable PDAC posed a significant challenge for detection, particularly in EV-DNA, where analysis was unsuccessful due to insufficient DNA yield. Third, the mutation analysis in cfDNA was restricted to two hotspot variants—*KRAS* p.G12D and *TP53* p.R273H—which, while common, do not capture the full mutational heterogeneity of PDAC and may underestimate the presence of other clinically relevant mutations.

While next-generation sequencing offers comprehensive molecular profiling and is increasingly applied in the context of precision medicine in PDAC, it is not always feasible for routine clinical use due to higher cost, longer turnaround time, and the need for specialized bioinformatics infrastructure. On the contrary, PCR-based methods such as castPCR and dPCR provide rapid, highly sensitive, and cost-effective detection of well-characterized hotspot mutations, with potential applications in perioperative treatment guidance or treatment monitoring. Our study presents a direct comparison of two fast and sensitive PCR-based technologies—castPCR and dPCR—for detecting clinically relevant somatic mutations in matched primary tumor and plasma samples from patients with resectable PDAC, offering a practical evaluation of their relative performance in a real-world clinical setting. Furthermore, by including a homogeneous cohort of patients undergoing upfront surgical resection, the study minimizes treatment-related effects and highlights the challenges of liquid biopsy in early-stage PDAC. Overall, the findings provide valuable insights into the utility and limitations of PCR-based approaches for perioperative molecular profiling.

In conclusion, our study demonstrates that both castPCR and dPCR are effective for detecting key somatic mutations in PDAC tumor tissue, with dPCR offering superior sensitivity, particularly in samples with low mutant allele frequency. The application of dPCR to liquid biopsy further highlights its potential as a minimally invasive tool for molecular profiling in the preoperative setting. Although ctDNA detection remains challenging in early-stage PDAC due to its low abundance, our findings support the integration of highly sensitive approaches such as dPCR to complement traditional tissue-based diagnostics. Future studies with larger cohorts, expanded mutation panels, and longitudinal sampling are warranted to validate these findings and to explore the clinical utility of liquid biopsy in guiding treatment decisions and monitoring disease progression in PDAC.

## 4. Materials and Methods

### 4.1. Patients and Sample Collection

Patients with preoperatively confirmed or suspected diagnosis of PDAC who underwent surgical resection at the University Medical Centre Ljubljana (Ljubljana, Slovenia) between 1 January 2018 and 31 December 2019 were eligible for study enrollment. All enrolled patients fulfilled the following eligibility criteria: (a) histopathologic diagnosis of PDAC; (b) scheduled for an upfront resection after discussion at a multidisciplinary team meeting; (c) no prior history of other cancers. FFPE tissue sections were prepared from resected specimens. Blood samples were prospectively collected immediately before the start of the surgical procedure, prior to any treatment.

The study was approved by the Republic of Slovenia National Medical Ethics Committee (Study No. 0120-155/2016-2, KME 106/03/16) and conducted in accordance with the Declaration of Helsinki. Written informed consent was obtained from all eligible patients prior to their enrollment.

### 4.2. Sample Processing and DNA Extraction

Tumor and normal tissue DNA were extracted from FFPE sections after punch biopsies of previously marked regions using the Maxwell RSC FFPE plus DNA purification kit (Promega Corporation, Madison, WI, USA) according to the manufacturer’s protocol. Extracted DNA was quantified using the Qubit 3.0 fluorometer (Thermo Fisher Scientific, Waltham, MA, USA).

Blood samples were collected in K2-EDTA collection tubes (6 mL) and processed by centrifugation at 2500× *g* for 10 min at 4 °C within four hours after collection. Plasma aliquots were stored at –80 °C until further use.

Up to 3.0 mL of plasma sample was used for the extraction of cfDNA using the PME free-circulating DNA Extraction Kit (iST Innuscreen GmbH, Berlin, Germany) following the manufacturer’s instructions. Extracted DNA was quantified spectrophotometrically using the LAMBDA Bio+ (PerkinElmer, Waltham, MA, USA).

As an alternative source of circulating DNA, extracellular vesicles were isolated from stored plasma samples for a subset of ten patients using the sucrose cushion ultracentrifugation method. Briefly, 1 mL of plasma was thawed on ice and centrifuged at 10,000× *g* for 20 min at 4 °C. Supernatant was diluted to 9 mL with phosphate-buffered saline and pipetted over 2 mL of 20% sucrose in 13 mL tubes. After centrifugation at 100,000× *g* for 2 h and 15 min at 4 °C (MLA-55 in Optima MAX-XP, Beckman Coulter, Brea, CA, USA), the supernatant was aspirated, the pellet suspended in 60 μL of phosphate-buffered saline, and the aliquots stored at –20 °C. The described procedure enables isolation of small extracellular vesicles, as extensively described by Holcar et al. [26].

The extraction of EV-DNA was performed using the QIAamp DNA Micro Kit (Qiagen, Hilden, Germany) according to the manufacturer’s instructions. Extracted DNA quantity was assessed by the LAMBDA Bio+ spectrophotometer (PerkinElmer, Waltham, MA, USA).

### 4.3. Analysis of Mutation Status

**Figure 5:**
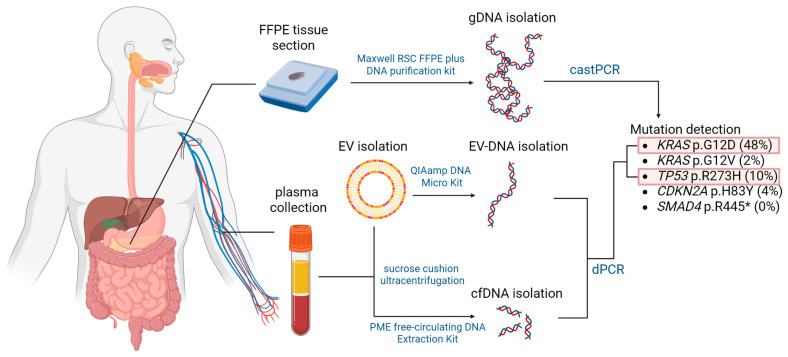
Schematic representation of the experimental workflow. DNA was extracted from primary tumor and plasma samples and subsequently assessed with castPCR and dPCR for the presence of selected mutations. FFPE—Formalin-fixed paraffin-embedded; EV—extracellular vesicles; gDNA—genomic DNA; EV-DNA—extracellular vesicle-associated DNA.

Isolated DNA samples were subjected to castPCR and dPCR analysis for the presence of common mutations in four PDAC driver genes, namely *KRAS*, *TP53*, *SMAD4,* and *CDKN2A*. Mutations were selected from the COSMIC (Catalogue of Somatic Mutations in Cancer) database based on their frequency in PDAC patients and are listed in Table 6. The workflow is summarized in Figure 5.

#### 4.3.1. castPCR Analyses

TaqMan Mutation Detection Assays with castPCR technology were used to analyze the prevalence of all selected mutations in DNA samples extracted from FFPE sections of PDAC. The castPCR technology enables high specificity and sensitivity of somatic mutation detection by using a combination of a mutant allele-specific primer and a wild-type allele-specific blocker within the same PCR reaction. The molecular blocker inhibits amplification of the wild-type allele, thereby enabling selective and efficient amplification of the mutant allele without interference. This enables the detection and quantification of low amounts of mutant allele in a sample that contains large amounts of normal, wild-type allele-containing DNA [27].

CastPCR analyses for *KRAS* p.G12D, *KRAS* p.G12V, *TP53* p.R273H, *CDKN2A* p.H83Y, and *SMAD4* p.R445* were performed using 20 ng of DNA per sample. Each PCR reaction consisted of 5 μL of 2× TaqMan Genotyping Master Mix (Life Technologies, Foster City, CA, USA), 1 μL of 10× TaqMan Mutation Detection Assay (Life Technologies, Foster City, CA, USA), 3 μL of nuclease-free water (Ambion, Invitrogen, Austin, TX, USA), and 1 μL of each DNA sample in a final volume of 10 μL. The assays were conducted on a 384-well plate using the QuantStudio 7 Flex Real-Time PCR System (Applied Biosystems, Thermo Fisher Scientific, Waltham, MA, USA) with the following thermal cycling conditions: initial denaturation at 95 °C for 10 min, followed by 5 cycles at 92 °C for 15 s and 58 °C for 1 min, and thereafter 40 cycles at 92 °C for 15 s and 60 °C for 1 min.

The results of the mutation detection assays were analyzed using Mutation Detector Software version v2.0 (Life Technologies, Foster City, CA, USA). The mutations were qualitatively assessed as either detected or not detected within a sample. For *KRAS* p.G12D, the calibration ΔC_T_ value for the mutant allele and corresponding gene reference assay was determined by the manufacturer, which enabled the calculation of the percentage of mutations present in a mutation-positive sample. DNA samples from normal pancreatic tissue were used as a reference to calculate the detection ΔC_T_ cutoff value for mutations for which no validated assay set with a predetermined detection ΔC_T_ cutoff value was provided by the manufacturer.

#### 4.3.2. dPCR Analyses

dPCR analysis relies on compartmentalization of the bulk PCR reaction into thousands of smaller independent reactions. After amplification, positive and negative reactions are counted to provide absolute quantification, offering enhanced accuracy and precision—particularly valuable for low-concentration samples.

We performed dPCR analysis for two somatic mutations, *KRAS* p.G12D and *TP53* p.R273H, as they were most commonly detected with castPCR in tumor DNA samples. *KRAS* p.G12D and *TP53* p.R273H were also analyzed in cfDNA isolated from preoperative plasma samples, while *KRAS* p.G12D was analyzed only in a subset of ten EV-DNA samples due to low DNA levels.

For dPCR analyses, DNA samples from tumor tissue were diluted to 7 ng/μL of gDNA. For cfDNA isolated from plasma, dilution was performed only for samples with a DNA concentration exceeding 40 ng/μL. No dilution was applied to extracted EV-DNA samples as they contained low amounts of EV-DNA.

Each PCR reaction consisted of 2.0 μL of Absolute Q DNA Digital PCR Master Mix (5×) (Thermo Fisher Scientific, Waltham, MA, USA), 0.25 μL of Absolute Q dPCR Assay (40×) (Thermo Fisher Scientific, Waltham, MA, USA), 2.75 μL of nuclease-free water (Ambion, Invitrogen, Austin, TX, USA), and 5 μL of DNA sample in a total volume of 10 μL. The assembled reaction mix was centrifuged at 10,000× *g* for 1 min and then pipetted in a volume of 9 μL to the first stop of each well on a QuantStudio Absolute Q Microfluidic Array Plate (MAP16) (Thermo Fisher Scientific, Waltham, MA, USA). Subsequently, 15 μL of Absolute Q Isolation Buffer (Thermo Fisher Scientific, Waltham, MA, USA) was loaded on top of the reaction mix in each well. Gaskets were applied across all units of the plate, and the plate was loaded onto the QuantStudio Absolute Q Digital PCR System (Thermo Fisher Scientific, Waltham, MA, USA), where compartmentalization, thermal cycling, and data collection were performed. The PCR thermal parameters were defined as follows: preheat step at 96 °C for 10 min, followed by 40 cycles at 96 °C for 5 s and 60 °C for 15 s.

Data were analyzed via QuantStudio Absolute Q software (version 6.2.1) (Thermo Fisher Scientific, Waltham, MA, USA). Normal tissue DNA was used to determine the thresholds of FAM and VIC signals. The number of FAM-positive (mutant) micro-chambers and mutant copies per μL was stated. The %mutation was calculated based on the following equation:

$$\%mutation=\;\frac{mutant\;copies\;per\;\mu L}{wild-type\;copies\;per\;\mu L}\;\cdot 100$$

A minimum of 200 wild-type signals per sample was required for a reaction to be considered valid.

We then assessed the mutation status of the *KRAS* and *TP53* genes in cfDNA samples isolated from blood samples obtained just before operating. Apart from sample dilution, the same steps were performed to assess the presence of *KRAS* p.G12D and *TP53* p.R273H mutations in cfDNA and *KRAS* p.G12D in EV-DNA. EV-DNA extraction and analysis were performed in a subset of ten patients only. Due to a low number of extracted EV-DNA copies, mutation detection was not successful, and further analysis of additional samples was not pursued.

### 4.4. Statistical Analysis

Continuous variables were described using median and interquartile range (25–75%, IQR), while categorical variables were described using frequencies. For continuous variables, normality of the distribution was assessed using the Shapiro–Wilk test. As the data were not all normally distributed, non-parametric tests were used for further analyses. Spearman’s rho correlation coefficient was used to evaluate correlations between continuous variables. Fisher’s exact test was used to compare the distributions of categorical variables. All statistical tests were two-sided, and the level of significance was generally set to 0.05. Statistical analyses were performed using IBM SPSS Statistics, version 27.0 (IBM Corporation, Armonk, NY, USA). Figures were prepared using Microsoft Excel and GraphPad Prism, version 10.5.0 (GraphPad Software, La Jolla, CA, USA).

## 5. Conclusions

Our study shows that both castPCR and dPCR effectively detect key somatic mutations in PDAC tumor tissue, with dPCR offering superior sensitivity. Despite challenges due to low ctDNA levels in early-stage PDAC, the enhanced sensitivity of dPCR enables its application to liquid biopsy samples, making it a promising minimally invasive complement to standard diagnostic methods.

## Figures and Tables

**Table 1:** Patients’ characteristics.

Characteristics		Total (N = 50)
Sex	Male, N (%)	27 (54.0)
	Female, N (%)	23 (46.0)
Age	Years, median (25–75%)	70 (61.0–77.0)
Tumor stage	1, N (%)	7 (14.0)
	2, N (%)	26 (52.0)
	3, N (%)	14 (28.0)
	4, N (%)	3 (6.0)
Tumor differentiation	Poor/moderately poor, N (%)	25 (53.2) [3]
	Moderately/moderately well, N (%)	20 (42.6)
	Well, N (%)	2 (4.3)
Metastasis	No, N (%)	47 (94.0)
	Yes, N (%)	3 (6.0)
Additional organ or vascular resection	No, N (%)	31 (62.0)
	Yes, N (%)	19 (38.0)
Adjuvant chemotherapy	No, N (%)	13 (26.5)
	Yes, N (%)	36 (73.5)
PFS	Months, median (25–75%)	11.8 (4.3–25.1) [1]
OS	Months, median (25–75%)	20.3 (8.3–44.0)
Follow-up	Months, median (25–75%)	64.5 (59.7–65.9)

The number of missing data is stated in [] brackets. OS: overall survival; PFS: progression-free survival.

**Table 2:** Frequency of genetic mutations detected in tumor DNA samples using competitive allele-specific TaqMan PCR (castPCR) (N = 50).

Genetic Mutation	COSMIC ID	Frequency, N (%)
*KRAS* p.G12D (c.35G>A)	521	24 (48.0)
*KRAS* p.G12V (c.35_36GT>TC)	515	1 (2.0)
*TP53* p.R273H (c.818G>A)	10660	5 (10.0)
*CDKN2A* p.H83Y (c.247C>T)	12504	2 (4.0)
*SMAD4* p.R445* (c.1333C>T)	14096	0 (0.0)

COSMIC: Catalogue of Somatic Mutations in Cancer.

**Table 3:** Frequency of *KRAS* p.G12D and *TP53* p.R273H mutations in primary tumor and plasma samples assessed by digital PCR (dPCR).

Mutation	Sample	N	% Mutations in All Patients, Median (25–75%)	% Mutations in Mutation Carriers, Median (25–75%)	>0%, N (%)	% Mutations in All Patients (Positive if >0.1%), Median (25–75%)	% Mutations in Mutation Carriers (Positive if >0.1%), Median (25–75%)	>0.1%, N (%)	>1%, N (%)
*KRAS* p.G12D	Primary tumor	49	14.3 (0.1–26.0)	15.2 (0.2–26.2)	47 (95.9)	14.3 (0.1–26.0)	16.7 (10.9–34.5)	37 (75.5)	35 (71.4)
	cfDNA	49	0.0 (0.0–0.1)	0.1 (0.0–0.2)	16 (32.7)	0.0 (0.0–0.0)	0.2 (0.2–0.2)	5 (10.2)	0 (0.0)
*TP53* p.R273H	Primary tumor	48	0.1 (0.0–0.1)	0.1 (0.1–0.1)	45 (93.8)	0.0 (0.0–0.1)	0.1 (0.1–0.2)	23 (47.9)	0 (0.0)
	cfDNA	49	0.0 (0.0–0.0)	0.0 (0.0–0.1)	4 (8.2)	0.0 (0.0–0.0)	*	1 (2.0)	0 (0.0)

* Too few patients (≤3). cfDNA: cell-free DNA.

**Table 4:** Number of patients with detected *KRAS* p.G12D mutations using castPCR and dPCR analysis. Reactions in dPCR analysis were considered positive when the %mutation exceeded 0% or 0.1%, as indicated.

		castPCR		
Cutoff	dPCR	-	+	*p*-Value
>0%	-	2 (7.7)	0 (0.0)	0.491 *
	+	24 (92.3)	23 (100.0)	
>0.1%	-	12 (46.2)	0 (0.0)	<0.001 *
	+	14 (53.8)	23 (100.0)	

* Fisher’s exact test; +—mutation detected; -—mutation not detected.

**Table 5:** Number of patients with detected *TP53* p.R273H mutations using castPCR and dPCR analysis. Reactions in dPCR analysis were considered positive when the %mutation exceeded 0% or 0.1%, as indicated.

		castPCR		
Cutoff	dPCR	-	+	*p*-Value
>0%	-	2 (4.7)	1 (20.0)	0.286 *
	+	41 (95.3)	4 (80.0)	
>0.1%	-	24 (55.8)	1 (20.0)	0.180 *
	+	19 (44.2)	4 (80.0)	

* Fisher’s exact test; +—mutation detected; -—mutation not detected.

**Table 6:** Selected mutations in pancreatic ductal adenocarcinoma (PDAC) driver genes.

Gene	COSMIC ID	Coding Sequence Mutation	Amino Acid Change
*KRAS*	521	c.35G>A	p.G12D
	515	c.35_36GT>TC	p.G12V
*TP53*	10660	c.818G>A	p.R273H
*SMAD4*	14096	c.1333C>T	p.R445*
*CDKN2A*	12504	c.247C>T	p.H83Y

## Data Availability

The data presented in this study are available on request from the corresponding author due to ethical reasons.

## Abbreviations

The following abbreviations are used in this manuscript:

PDAC	Pancreatic ductal adenocarcinoma
cfDNA	Cell-free DNA
ctDNA	Circulating tumor DNA
EV-DNA	Extracellular vesicle-associated DNA
castPCR	Competitive allele-specific TaqMan PCR
dPCR	Digital PCR
FFPE	Formalin-fixed paraffin-embedded
COSMIC	Catalogue of Somatic Mutations in Cancer
